# Size‐Dependent Ultrasound Activation of Thrombin Catalytic Activity by Mechano‐Nanoswitches

**DOI:** 10.1002/advs.202510707

**Published:** 2025-11-26

**Authors:** Menghan Xiao, Zhihuan Liao, Junliang Chen, Xiong Zuo, Jiangting Zeng, Xiangfu Du, Zihao Teng, Johannes Hahmann, Andreas Herrmann, Shuaidong Huo

**Affiliations:** ^1^ State Key Laboratory of Vaccines for Infectious Diseases Xiang An Biomedicine Laboratory Fujian Provincial Key Laboratory of Innovative Drug Target Research School of Pharmaceutical Sciences Xiamen University Xiamen 361102 China; ^2^ Jiangxi Provincial Key Laboratory of Natural Biomimetic Drug Research College of Pharmacy Jiangxi Normal University Nanchang 330022 China; ^3^ DWI – Leibniz Institute for Interactive Materials 52056 Aachen Germany; ^4^ Institute of Technical and Macromolecular Chemistry RWTH Aachen University 52074 Aachen Germany; ^5^ Max Planck School Matter to Life 69120 Heidelberg Germany

**Keywords:** enzyme activation, nano‐mechanochemistry, nanoswitch, size‐dependence, ultrasound

## Abstract

Integrating nanostructures with mechanochemistry significantly boosts drug loading capabilities and sonomechanical drug activation, outperforming traditional polymer systems. Nevertheless, the influence of nanoparticles’ physicochemical properties on their mechanochemical responses remains largely unexplored. In this work, different‐sized mechano‐nanoswitches consisting of two gold nanoparticles (5, 10, and 20 nm), which are bridged by a single DNA strand that is harboring a thrombin binding site loaded with the enzyme, are constructed to investigate the relationship between size and force responsiveness under ultrasound. When subjected to ultrasonication, these nanoswitches are stretched and unfolded, triggering the release and catalytic activation of thrombin. The results demonstrate that the largest nanoswitches (20 nm) exhibit the most pronounced ultrasound responsiveness, reaching up to two times that of the smallest nanoswitches (5 nm). This investigation deepens the basic understanding of the critical role of the size of colloidal nanoparticles in mechanochemistry and offers valuable insights for developing novel mechanoresponsive nanostructures, paving the way for more efficient mechanically controlled drug or protein activation strategies.

## Graphical abstract

The fundamental principles linking nanoparticle (NP) physicochemical properties to their interactions with sonomechanical forces are not well‐defined. This work systematically investigates the size‐dependent ultrasound activation of thrombin using different‐sized mechano‐nanoswitches, which provides critical insights into the role of NP size in nano‐mechanochemistry, laying a foundation for designing advanced nanosystems for the precise, mechano‐regulated control of therapeutic activity.

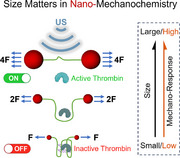

## 1 Introduction

Mechanochemistry, which leverages macroscopic forces to trigger microscopic molecular responses, has recently seen significant advancements in biomedical applications.^[^ ^1^, ^2^, ^3^ ^]^ In polymer mechanochemistry, ultrasound (US)‐induced cavitation is employed to drive specific chemical transformations via mechanophores embedded within polymer matrices.^[^ ^4^, ^5^ ^]^ Beyond the precise design of mechanophores, the structure of the polymer backbone plays a crucial role in determining their response to sonomechanical forces.^[^ ^6^, ^7^ ^]^ For decades, the structure‐activity relationship of polymers, including factors such as contour length, degree of polymerization, and polymer conformation, has been extensively studied and is well understood in the context of polymer mechanochemistry.^[^ ^8^, ^9^, ^10^ ^]^

Building on these foundational principles, polymer mechanochemistry has opened new possibilities for altering drug activity,^[^ ^11^ ^]^ exemplified by our first demonstration of ultrasound‐induced mechanochemical bond scission for drug activation.^[^ ^12^ ^]^ Furthermore, we pioneered the integration of nanoparticle (NP) systems with mechanochemistry –nano‐mechanochemistry, enabling controlled drug release and enzyme activation.^[^ ^12^, ^13^, ^14^ ^]^ This approach significantly enhances mechanical responsiveness and drug‐loading capacities compared to conventional polymer systems.

NPs, characterized by their functionalizable surfaces and customizable structures, provide a facile and versatile platform for studying mechanochemical behavior.^[^ ^15^, ^16^ ^]^ However, the fundamental principles underlying nano‐mechanochemistry are still not well‐defined. A key issue is whether and how the physicochemical characteristics of NPs affect their interaction with sonomechanical forces at the nanoscale. Additionally, it remains uncertain how the established concepts of polymer mechanochemistry can be referenced or adapted for nano‐mechanochemical studies.

**Scheme 1:**
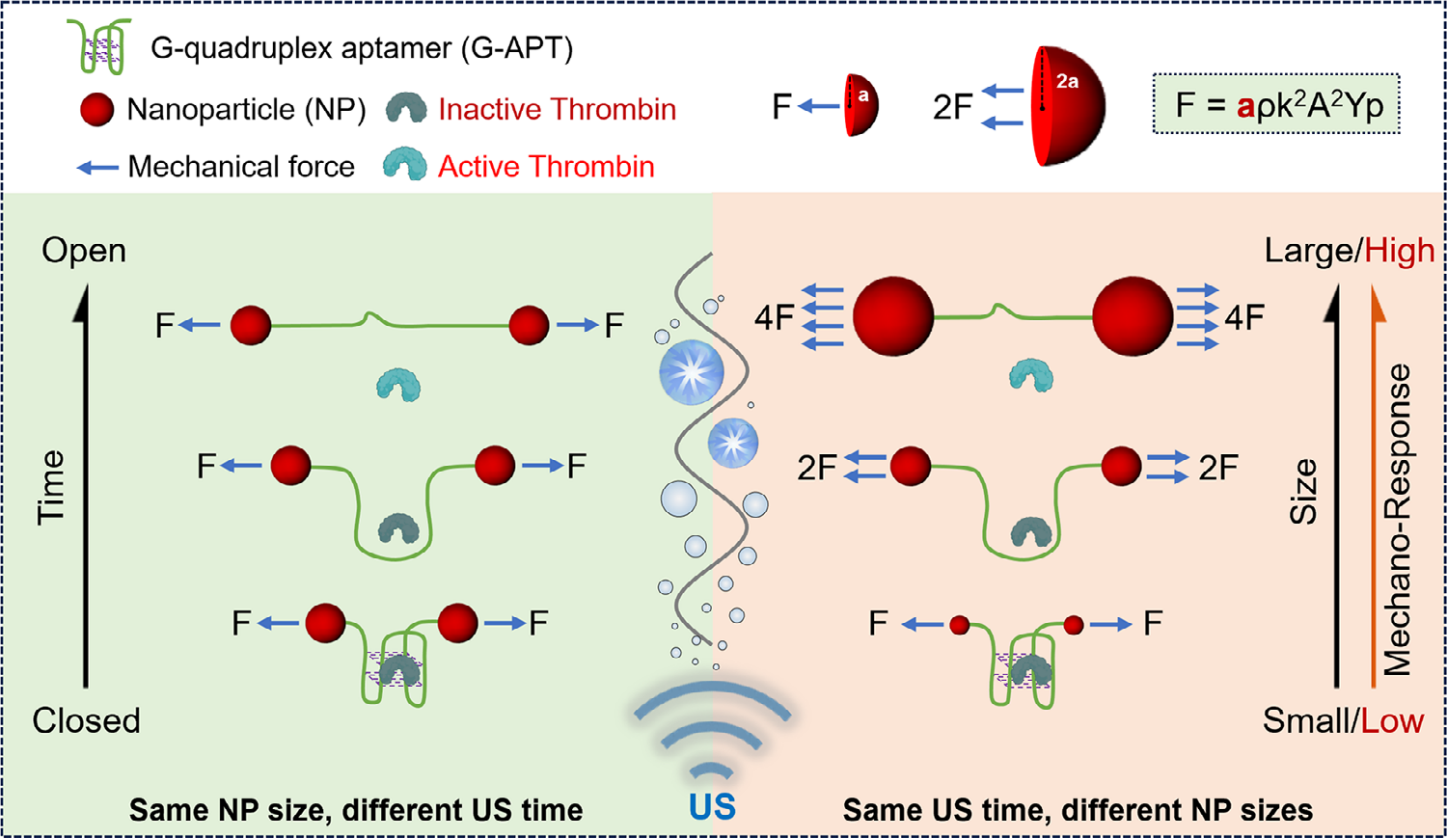
Size‐dependent ultrasound activation of the thrombin catalytic activity by mechano‐nanoswitches.

Size is undoubtedly one of the most critical factors governing the properties and behavior of NPs in various applications.^[^ ^17^ ^]^ To deepen our understanding of how NP size affects their mechanical force responses, we constructed mechano‐nanoswitches using gold nanoparticle (AuNPs) dimers of varying sizes, allowing us to systematically investigate the impact of NP size on sonoresponsiveness and its subsequent effect on catalytic activity (**Scheme** **1**). We selected thrombin (Th) as a model protein that catalyzes the formation of fibrin from fibrinogen, given that its activity is readily monitored through light scattering.^[^ ^18^, ^19^ ^]^ For the construction of nanoswitches, we employed the G‐quadruplex aptamer (G‐APT) as a linker to assemble AuNP dimers (Au─G─Au) with sizes of 5, 10, and 20 nm.^[^ ^20^ ^]^ The robust binding of G‐APT to Th allowed us to capture and deactivate Th by forming Au─Th─Au nanoswitches. Upon ultrasonication, the folded G‐APT structure was mechanically stretched, disrupting the specific non‐covalent interactions between Th and G‐APT. This mechanical cleavage led to the release and catalytic activation of Th. Notably, the efficiency of release was found to be directly correlated with the size of AuNPs. As the size increased, more nanodimers were pulled apart, and more Th was released and thus activated, with the 20 nm Au─Th─Au nanodimers showing the highest efficiency. A 1‐min sonication session was sufficient to restore ≈ 70% of the catalytic activity, which was 1.5 times higher than that of the 10 nm dimers and twice that of the 5 nm dimers. These findings underscore the critical influence of NP size on their sonoresponsiveness and catalytic activation. This study represents an important step toward understanding the pivotal role of NPs’ physicochemical properties in nano‐mechanochemistry. By establishing size as a key parameter in designing mechano‐responsive systems, this work provides foundational insights that will guide the development of more efficient nanosystems for mechano‐based precise drug activity regulation.

## 2 Results and Discussion

### 2.1 Preparation and Characterization of Au─G─Au Nanodimers with Different Sizes

**Figure 1:**
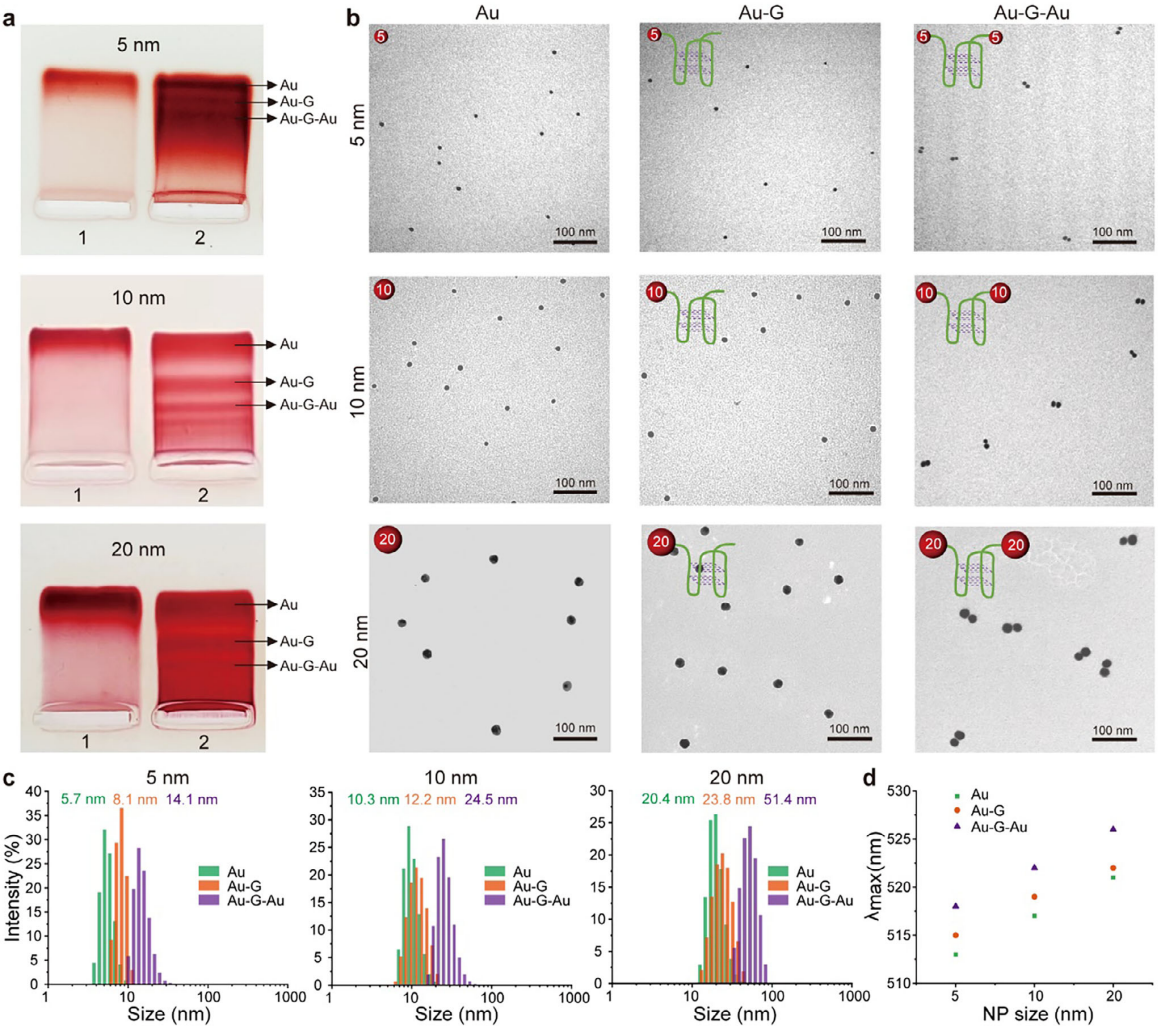
Purification and characterization of Au─G─Au nanodimers with different sizes. a) Electrophoretic analysis of G‐quadruplex‐bridged nanodimers linked by 5, 10, and 20 nm AuNPs. Lane 1: pristine AuNPs as a reference; Lane 2: Bands of AuNPs functionalized by G‐quadruplex after coupling. b) Representative TEM image of different‐sized Au, Au─G, and Au─G─Au purified by agarose gel electrophoresis. Scale bars: 100 nm. c) The hydrodynamic size analysis of Au, Au─G, and Au─G─Au of various sizes. d) Variation of λmax of different‐sized Au, Au─G, and Au─G─Au.

To explore the mechanical response behavior of different‐sized mechano‐nanoswitches under US, we first prepared 5, 10, and 20 nm AuNPs, as reported before.^[^ ^21^ ^]^ Then, G‐APTs were used as bridges to connect AuNPs to form different‐sized nanodimers. The G‐APT structure was obtained after annealing the terminally double‐thiolated TBA_15_ aptamer as reported before,^[^ ^22^ ^]^ and was confirmed by agarose gel electrophoresis (Figure S1, Supporting Information) and circular dichroism (Figure S2, Supporting Information). After conjugation, the different‐sized Au nanodimers bridged with a G‐APT (Au─G─Au) were finally isolated and recovered from the production mixtures by agarose gel electrophoresis. As shown in **Figure** **1**a, the separate bands display gradually slower mobility in the order of bare AuNPs (Au), single aptamer modified mono‐AuNP conjugates (Au─G), and Au─G─Au with different sizes, respectively. Transmission electron microscopy (TEM) images demonstrated the composition of each band as well as the high purity of Au─G─Au nanodimer production (Figure 1b). The nanodimer formation was further confirmed through dynamic light scattering (DLS) and UV–vis spectroscopy (Figure S3, Supporting Information). DLS measurements showed that the hydrodynamic size doubled compared to single particles (Figure 1c), while the plot of wavelength maximum (λmax) of these nanodimers showed a significant bathochromic shift (Figure 1d). Together, these results validate the successful assembly of Au─G─Au nanodimers.

### 2.2 Sonomechanical Response of Different‐Sized Au─G─Au Nanodimers

**Figure 2:**
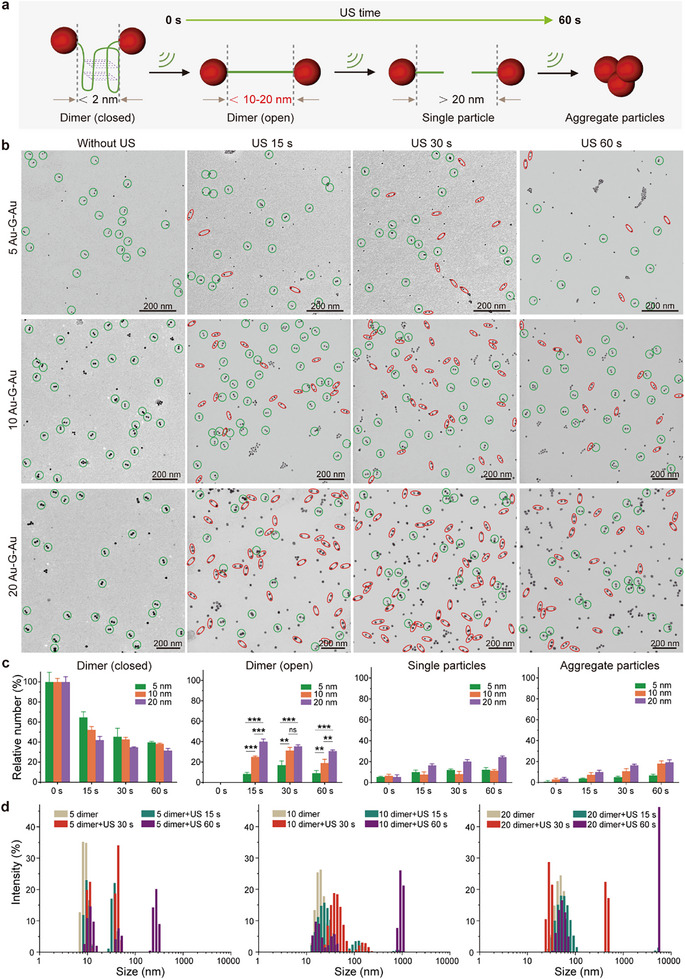
US‐induced structural change of different‐sized Au─G─Au nanodimers. a) Schematic diagram of ultrasonic manipulation and dimer changes under US. b) Representative TEM images of different‐sized Au─G─Au without US, Au─G─Au with US for 15, 30, and 60 s, respectively (green circled: closed; red circled: open). Scale bars: 200 nm. c) The relative number percentage histograms of different‐sized Au─G─Au with different morphologies of dimer (closed), dimer (open), single particles, and aggregated particles after US treatment for 0, 15, 30, and 60 s, respectively. The statistical analysis was repeated with three independent samples. Mean values ± standard deviation, N = 3 independent experiments. Statistical differences were determined by an one‐way ANOVA with Tukey's post hoc test, ns (not significant), ^**^ *p* < 0.01, ^***^ *p* < 0.001. d) The hydrodynamic size analysis of different‐sized Au─G─Au dimer with sonication irradiation for 0, 15, 30, and 60 s, respectively.

To verify our hypothesis of applying mechanical force to stretch the Au─G─Au nanodimer structures, we studied the US responsiveness of different‐sized Au─G─Au nanodimers (**Figure** **2**a). With the help of TEM, as shown in Figure 2b, we found that some of the initially closed nanodimers (green circled) changed to an open state with a sizeable interparticle gap (≈10–20 nm) after 15 s ultrasonication (red circled). Interestingly, only several 5 nm nanodimers were stretched apart after the same 15 s US treatment (20 kHz, 150 W, 2.0 s on, 1.0 s off at 50% Amplitude), while as the size increased to 20 nm, the number of open nanodimers significantly increased. These findings suggest a close relationship between the efficiency of nanodimer force‐stretching and their particle size. As expected, with the extension of sonication time, more open nanodimers could be observed for the same size. More importantly, the US‐induced structural changes became more pronounced as the size increased. The stretching of Au─G─Au nanodimers is explainable by the sonomechanical dissociation of the multi‐hydrogen bonding interactions within the G‐APT structure. Meanwhile, many single particles and aggregate formation were observed after prolonged sonication, most likely caused by covalent bond scission along the aptamer backbone.

To enhance the statistical significance of the data, more TEM images of different‐sized Au─G─Au nanodimers were recorded, measured, and statistically analyzed throughout the sonication process (Figure 2c). The application of US for 15 s reduced the closed state of 5 nm Au─G─Au nanodimers to ≈65%. For the larger 10 and 20 nm nanodimers, the percentage of closed dimers decreased to ≈52% and 40%, respectively, after the same sonication duration. As the sonication time extended, more closed dimers of different sizes were pulled apart, with the 20 nm dimers being reduced to only 30% remaining. At the same time, the number of opened nanodimers increased in a size‐dependent manner, with 15 s of sonication separating ≈40% of the 20 nm nanodimers, nearly twice the separation observed for the 10 nm dimers and four times that of the 5 nm dimers. These findings suggest that the 20 nm dimers possess the highest sonomechanical responsiveness. A 30 s sonication period also revealed that smaller dimers require a longer time to undergo stretching or covalent cleavage. As previously mentioned, prolonged sonication was accompanied by the appearance of single particles and aggregates, indicating that continuous ultrasonication further compromised the particle surface functionalization and colloidal stability. Additionally, DLS was employed to monitor changes in hydrodynamic properties (Figure 2d). Sonication caused the dynamic size distribution of all nanodimers to shift toward larger populations and gradually form large aggregates, aligning with the TEM observations and statistical analysis discussed above. Notably, the US‐triggered conformational change of the nanodimers exhibited partial reversibility. Taken 10 nm nanodimers as an example, the results show that the population of open dimers decreased by ≈15% over a 30‐min recovery incubation at room temperature, with a corresponding increase in the population of closed dimers. At the same time, the DLS investigation revealed a consistent shift in hydrodynamic size, indicating a partial reversion of the open dimers to their closed state via G‐quadruplex re‐folding (Figure S4, Supporting Information).

### 2.3 Thrombin Loading and US‐Induced Catalytic Activation of Different‐Sized Au─G─Au Nanodimers

**Figure 3:**
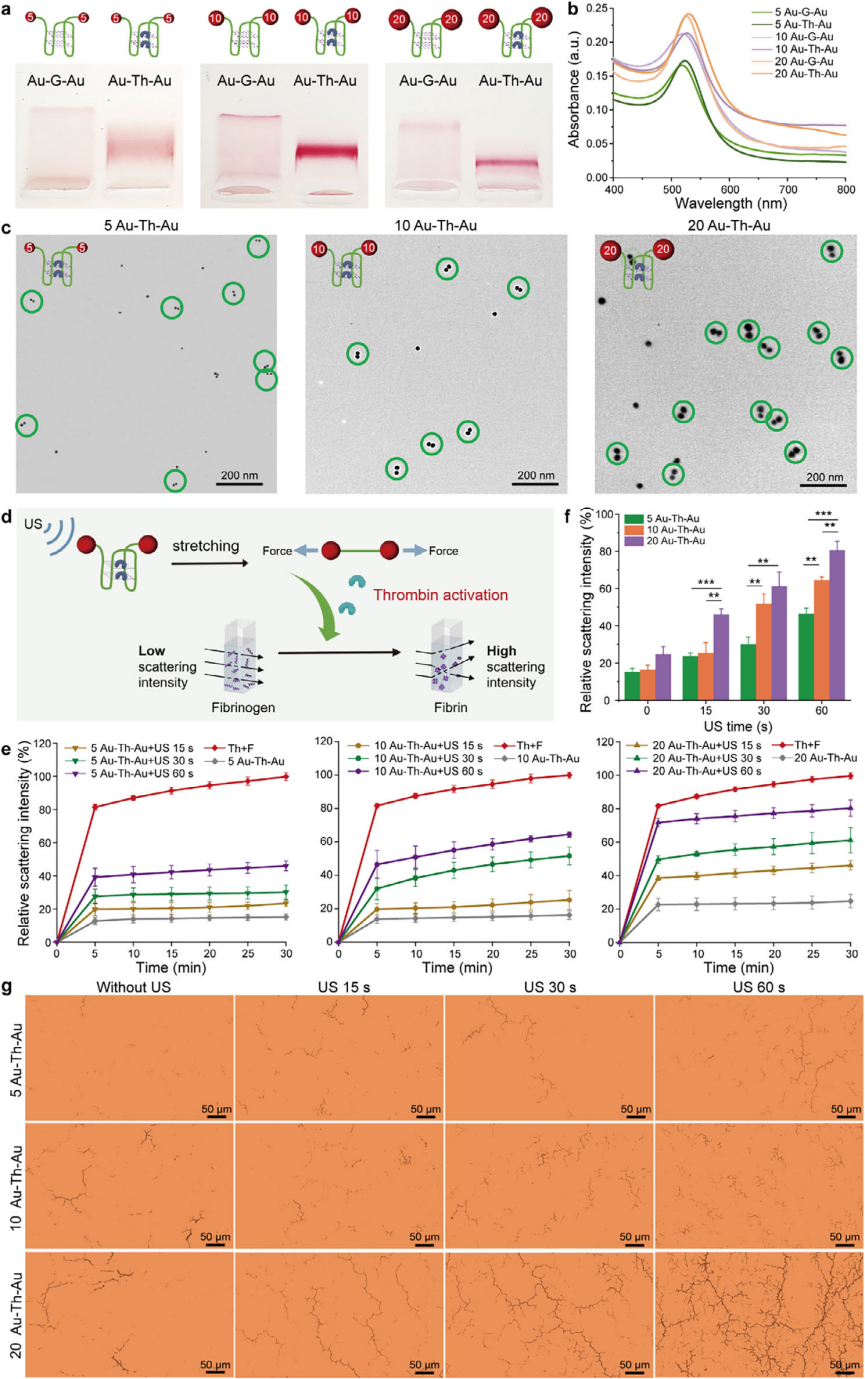
Loading and activation of thrombin (Th). a) Agarose gel (3%) characterization of Th loading on Au─G─Au of various sizes. b) UV–vis absorption spectra of different‐sized Au─G─Au after Th loading. c) Representative TEM images of Au─Th─Au of various sizes. Scale bars: 200 nm. d) Schematic diagram of scattering intensity detection of fibrin formation after applying US. e) Fibrinogen scattered light intensity signal after adding Th released by Au─Th─Au of different sizes after US treatment. f) Comparison of scattered light intensity of different nanoswitches at 30 min of coagulation. Mean values ± standard deviation, N = 3 independent experiments. Statistical differences were determined by an one‐way ANOVA with Tukey's post hoc test, ^**^ *p* <0.01, ^***^ *p* <0.001. g) Optical microscope images of fibrinogen treated with Th released by Au─Th─Au of different sizes after US treatment. Scale bars: 50 µm.

To further utilize and quantify the mechanical response efficiency of dimers with different sizes, we selected the enzyme Th that catalyzes fibrin formation from fibrinogen as a model protein for constructing Au─Th─Au nanoswitches. Based on the robust recognition and protein‐loading capacity of G‐APT, Th was captured and deactivated by forming a well‐defined aptamer‐protein complex.^[^ ^23^ ^]^ Before loading to Au─G─Au nanodimers, the selective binding property of Th with the annealed G‐APT was verified. Gel electrophoresis results revealed that more and more complexes were retained with the increase of Th during the run (Figure S5, Supporting Information). Meanwhile, G‐APT without annealing was used as a control, which showed no apparent binding with Th (Figure S6, Supporting Information). The successful loading of Th onto the different‐sized Au─G─Au nanodimers was confirmed using agarose gel electrophoresis. All three Au─Th─Au conjugates displayed reduced mobility compared to their corresponding Au─G─Au dimers (**Figure** **3**a). The bathochromic shift of the UV–vis absorption spectra of different‐sized Au─Th─Au further confirmed the successful loading of Th on the nanodimers (Figure 3b). Moreover, TEM showed no visible change to the closed structure of different‐sized nanodimers upon Th loading (Figure 3c).

Subsequently, we investigated the release and activation of Th from different‐sized Au─Th─Au nanodimers with mechanical force. Scattered light assays were used to monitor the conversion of fibrinogen to fibrin, revealing that Th molecules were successfully released from different‐sized nanodimers (Figure 3d). As expected, longer sonication times lead to a higher scattering intensity, indicating a higher fibrinogen concentration (Figure 3e). More importantly, a pronounced size‐dependent enhancement in sonoactivated catalytic efficiency was observed, with the 20 nm Au─Th─Au nanodimers exhibiting the highest performance. Notably, a mere 60 s of sonication activated ≈ 80% of the catalytic activity for the 20 nm constructs, achieving levels 1.5‐ and 2‐fold greater than the 10 and 5 nm nanodimers, respectively (Figure 3f). This superior responsiveness was further corroborated by kinetic analysis of the coagulation process, wherein the 20 nm nanodimers also demonstrated significantly higher initial rate constants (within the first 5 min) and average reaction rates compared to their smaller counterparts (Figure S7, Supporting Information). At the same time, annealed pristine G‐APT without NP functionalization, combined with Th (G‐APT‐Th) were sonicated as controls, which showed only slight scattered light intensity under US, demonstrating the importance and necessity of the Au conjugation to achieve US responsiveness. In addition, the US shows little influence on the catalysis activity of Th itself upon the same sonication duration (Figure S8, Supporting Information). Furthermore, we used optical microscopy to verify the US‐activated fibrin formation. As shown in Figure 3g, the gradually formed fibrous networks proved the catalytic formation of fibrin via force‐induced release of Th. Again, the microscopy pictures of fibrin fibers demonstrate the size effect of nanodimers. As shown by similar control experiments as above, the Au nanodimers are needed to achieve US‐responsiveness, while free Th is neglectable affected by sonication (Figure S9, Supporting Information).

### 2.4 Au─Th─Au Nanoswitches‐Based Catalyst for US‐Controlled Catalytic Reaction

**Figure 4:**
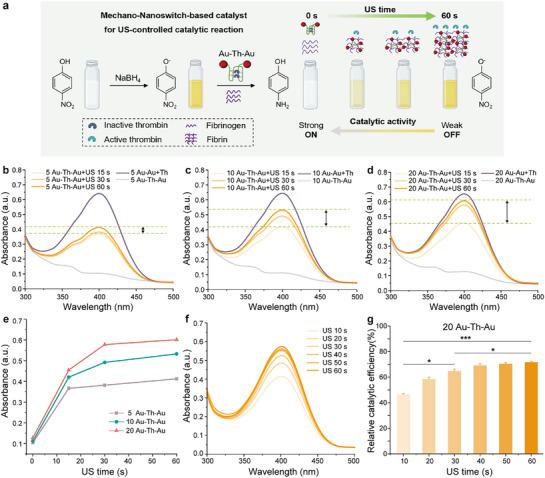
US‐controlled catalysis of 4‐nitrophenol under the action of the US within different‐sized Au─Th─Au. a) Schematic representation of the reduction reaction at different ultrasonic times of the nanoswitches‐based catalyst. b–d) Absorption spectra and color change of 4‐nitrophenol at different sonication times for each size of Au─Th─Au. b) 5, c) 10, and d) 20 nm. e) Comparison of the effect of Au─Th─Au of different sizes on the absorption spectra at 400 nm of 4‐nitrophenol. f) Absorption spectra and color change of 4‐nitrophenol at various ultrasonic times of 20 nm Au─Th─Au. g) Relative catalytic efficiency of 20 nm Au─Th─Au after various times of sonication. Mean values ± standard deviation, N = 3 independent experiments. Statistical differences were determined by an one‐way ANOVA with Tukey's post hoc test, ^*^ *p* <0.05, ^***^ *p* <0.001.

The catalytic reduction of 4‐nitrophenol serves as a model reaction to test the catalytic activity of metal NPs in aqueous solution.^[^ ^24^, ^25^ ^]^ Once metal NPs are added, the catalytic process follows first‐order kinetics, and the solution gradually changes from yellow to colorless since 4‐aminophenol is formed. The strong absorption of 4‐nitrophenolate ions at 400 nm can be readily monitored by UV–vis spectroscopy.^[^ ^26^ ^]^ Encouraged by the US‐activated catalytic capacity of Au─Th─Au nanodimers demonstrated above, we attempted to control the catalytic process of 4‐nitrophenol using the different‐sized mechano‐nanoswitches (**Figure** **4**a).

First, the conversion of 4‐nitrophenol with sodium borohydride was catalyzed by nanodimers, as indicated by a color change from yellow to colorless (Figure S10, Supporting Information). Then, we explored the size dependency of Au─Th─Au nanoswitches regarding the catalytic transformation of yellow 4‐nitrophenol to colorless 4‐aminophenol. The pristine G‐APT‐Th was used as a control experiment that cannot release Th due to the lack of AuNPs, resulting in the catalytic reaction (Figure S11, Supporting Information). The addition of free Th catalyzed fibrinogen to insoluble fibrin, which encapsulated the nanodimers and, therefore, inhibited the catalytic reaction with 4‐nitrophenol (Figure S12, Supporting Information). As expected, the absorbance is independent of sonication time in this case. In contrast, Au─Th─Au mechano‐nanoswitches resulted in varying degrees of inhibition of the catalytic process depending on sonication time (Figure 4a). Fibrin only formed when activated by US to prevent AuNPs from catalyzing the formation of 4‐aminophenol, which could also be controlled arbitrarily by adjusting sonication time (Figure 4b–e). Importantly, these results showed that different‐sized Au─Th─Au nanoswitches exhibited distinct regulatory abilities for the catalytic process. Compared to the 5 nm Au─Th─Au nanoswitches, the 20 nm counterparts demonstrated a broader control threshold and higher sensitivity under 60 s of sonication. Their relative catalytic performance could be controlled over a range of up to 70% within this timeframe, highlighting the potential of Au─Th─Au mechano‐nanoswitches for ultrasound‐controlled catalytic reactions (Figure 4f,g). These results further underscore the critical role of NP size in determining their sonomechanical behavior.

In recent decades, extensive research has been conducted on the mechanical impact of acoustic fields on the scission of polymer chains in solution.^[^ ^27^, ^28^ ^]^ It has been widely acknowledged that the solvodynamic forces generated by ultrasonic cavitation are most intense at the center of the polymer chain, leading to the establishment of the center cleavage model.^[^ ^29^, ^30^ ^]^ Similarly, the “stretching and breakage” mechanism also adapts in nano‐mechanochemistry, while the NPs serve as the medium for force application and transmission.^[^ ^31^ ^]^ In this study, the observed sonomechanical response of nanodimers is correlated to the NP diameter. The most reasonable explanation for the pronounced liberation of Th from the 20 nm particle system compared to the smaller ones is that larger particles are more susceptible to cavitation and effectively transmit the generated force to cleave the weak bonds between Th and its aptamer.

According to the equation of the acoustic radiation force experienced by a solid sphere: *F = aρk^2^A^2^Yp*, where *a* is the particle radius, *ρ* is the density of the medium, *k* is the wavenumber of the medium, *A* is the ultrasound amplitude, and *Yp* is the force per unit cross‐section, which is a constant equal to 1.^[^ ^32^, ^33^ ^]^ When other factors remain unchanged, the sonomechanical force acting on the particles is linearly related to their radius or diameter. While a positive size‐force correlation is strongly supported within the 5–20 nm range, this trend may not be linear or monotonic at larger sizes. Considering experimental errors and other impact factors, such as reflection and refraction of ultrasound radiation at the NP interface, the experimental trend obtained in this study is generally consistent with the theoretical calculation, highlighting the critical law of size dependence in nano‐mechanochemistry.

## 3 Conclusion

In summary, for the first time, we explored the sonomechanical behavior of different‐sized nanodimers and found that their response is size‐dependent. By systematically comparing the mechanical responsiveness among nanodimers with varying sizes under US, we demonstrated that larger NPs significantly enhance the mechanochemical scission rates of weak bonds. This finding highlights the potential to optimize NP size to improve the efficiency of mechanical force transmission. Moreover, we proved that mechano‐nanoswitches are a versatile platform for the US‐induced liberation of bioactive payloads. These results underscored the pivotal role of nanoparticle size for the mechanical force conduction to mechanophores in nano‐mechanochemistry. By employing the US as a controllable external trigger, this approach paves the way for developing more efficient and well‐regulated drug activation nanosystems with significant potential for biomedical and therapeutic applications.

## Conflict of Interest

The authors declare no conflict of interest.

## Author Contributions

M.X. and Z.L. contributed equally to this work. M.X. and Z.L. conceived the idea and designed the research. M.X. synthesized and characterized the nanostructures and other experiments. M.X. and S.H. wrote the paper, and all other authors contributed to the discussion and preparation of the manuscript. S.H. and A.H. supervised all the studies described in this article.

## Supporting information



Supporting Information

## Data Availability

The data that support the findings of this study are available from the corresponding author upon reasonable request.
