# Supplementary material for: Size‐Dependent Ultrasound Activation of Thrombin Catalytic Activity by Mechano‐Nanoswitches
Source: Adv Sci (Weinh). 2025 Nov 26;13(7):e10707. doi: 10.1002/advs.202510707 (PMC12866693; doi:10.1002/advs.202510707)
Supplement: Supplementary file 1 — Supporting Information [file ADVS-13-e10707-s001.docx]

**Electronic Supplementary Information**

**for**

**Size-Dependent Ultrasound Activation of Thrombin Catalytic Activity by Mechano-Nanoswitches**

Menghan Xiao,^a,#^ Zhihuan Liao,^a,#^ Junliang Chen,^a^ Xiong Zuo,^b^ Jiangting Zeng,^a^ Xiangfu Du,^a^ Zihao Teng,^a^ Johannes Hahmann^c,d,e^, Andreas Herrmann^c,d,e^* and Shuaidong Huo^a,^*

^a^ State Key Laboratory of Vaccines for Infectious Diseases, Xiang An Biomedicine Laboratory, Fujian Provincial Key Laboratory of Innovative Drug Target Research, School of Pharmaceutical Sciences, Xiamen University, Xiamen 361102, China

^b^ Jiangxi Provincial Key Laboratory of Natural Biomimetic Drug Research, School of Healthy, Jiangxi Normal University, Nanchang 330022, China

^c^ DWI – Leibniz Institute for Interactive Materials, 52056 Aachen, Germany

^d^ Institute of Technical and Macromolecular Chemistry, RWTH Aachen University, 52074 Aachen, Germany

^e^ Max Planck School Matter to Life, 69120 Heidelberg, Germany

^#^ M.X. and Z.L. contributed equally to this work.

* Correspondence to: [huosd@xmu.edu.cn](mailto:huosd@xmu.edu.cn) and [herrmann@dwi.rwth-aachen.de](mailto:herrmann@dwi.rwth-aachen.de)

**Materials**

All chemical reagents were of analytical grade and were used without further purification unless otherwise stated. The oligonucleotide sequence TBA_15_ (TTTTTTTTTTTTTTTGGTTGGTGTGGTTGGTTTTTTTTTTTTTTT) was synthesized and HPLC-purified by Sangon Biotech Co., Ltd. (Shanghai, China). Chloroauric acid (HAuCl_4_), sodium citrate, and bis(p‑sulfonatophenyl)phenyl-phosphine dehydrate dipotassium (BSPP) were obtained from Sigma-Aldrich. Tris(2‑carboxyethyl) phosphine hydrochloride (TCEP·HCl) and DNA ladder were ordered from Sangon Biotech Co., Ltd. Thrombin and Fibrinogen were obtained from Yuanye Bio-Technology Co., Ltd. (Shanghai, China). Other chemicals were purchased from Sigma-Aldrich unless otherwise noted. Milli-Q water was used throughout all the experiments.

**Methods**

Preparation and synthesis of Au-G-Au nanodimer

5, 10, and 20 nm Au-Citrate NPs were prepared by the standard citrate reduction method. To obtain citrate-stabilized AuNPs of different sizes, a ligand exchange process was performed by adding bis(p-sulfonatophenyl)phenylphosphine (BSPP) as reported.^[1]^ Solid sodium chloride was then added to the above AuNPs solution until the color transitioned to a distinct blue hue. After 12 h of incubation at room temperature, the solution was centrifuged and resuspended in Milli-Q water. Au-G-Au dimers were prepared by mixing AuNPs with at both ends terminally thiolated G-quadruplex (TCEP treated) in a molar ratio of 4:1-2:1, and the mixture was incubated for 4-12 h in 0.5×TBE buffer (containing 50 mM NaCl). After that, the complex was centrifuged to remove excess free G-quadruplex.

G-quadruplex forming and thrombin loading

TBA_15_ (20 µM) in Tris-HCl binding buffer (pH 7.4, 100 mM KCl) was heated to 95°C for 5 min, then slowly cooled down to room temperature overnight to form the G‑quadruplex. For the thrombin loading, different concentrations of thrombin (10, 20, and 40 µM) were incubated with G-quadruplex, respectively, and the mixtures were kept at 37°C for 30 min.

Agarose gel electrophoresis

For the characterization of dimers, 3% agarose gel was used to determine the formation of Au-G-Au dimers as well as to isolate them. Electrophoresis was carried out at 13 V/cm for 20 min in 0.5× TBE running buffer. Finally, the Au-G-Au dimer band was cut from the gel to be purified in a dialysis membrane (MWCO 3000-10000) through electroelution. The binding of G-quadruplex with thrombin was demonstrated by agarose gel electrophoresis (4%). Electrophoresis was carried out in 1× TAE buffer at 90 V for 80 min.

Sonication experiments

Ultrasonication experiments at *f* = 20 kHz were performed with a SONICS VCX150 sonicator (USA) equipped with a 3 mm diameter microtip probe. Sonication was performed using pulsed ultrasound (2.0 s on, 1.0 s off at 50% Amplitude). The input power level was 150 W. The vessel was placed in an ice bath to maintain a temperature of 6-9°C inside the vessel throughout sonication.

Transmission electron microscopy (TEM) observation and analysis

Samples were prepared and dripped on a copper grid and dried at room temperature. A JEOL JEM-2100plus with 200 kV accelerating voltage was used to record TEM images. For studying the interparticle distance, several TEM images of different sizes of dimers were recorded, measured, and statistically analyzed after various sonication times. The distances of each particle with its contiguous particles were measured, marked and recorded. Since the length of each base in double-thiolated TBA_15_ is 0.34 nm^[2]^, so the theoretical length of G-quadruplex is calculated to be ca. 15 nm. 15 nm was used as the critical size to determine the dimer (open) and single particle. The observation and measurement were repeated with three independent samples.

Hydrodynamic diameter measurement and analysis

NPs were well diluted and dispersed in Milli-Q water before the experiments. The hydrodynamic diameter of the prepared AuNPs and Au-G-Au dimer structures pre- and post-sonication were measured by a Nano ZS Zetasizer (25°C, Malvern, England).

Scattered light monitoring

To explore the behavior of thrombin release and measure the activated thrombin from Au-Th-Au, fibrinogen conversion to fibrin was selected as a monitoring reaction. When fibrinogen was converted to fibrin by activated thrombin, its scattered light intensity increased. Firstly, different sizes of Au-Th-Au were treated by sonication with the probe immersed in the fluid for 15, 30 and 60 s. After US treatment, the reaction was tested by a 250 µL quartz fluorescence cuvette in 30 min. For monitoring scattering, the excitation and emission wavelengths were both 580 nm, and the emission was detected at a right angle relative to the light excitation so that the excitation light did not interfere with the light-scattering signal. In general, fibrinogen (7.8 mg/mL) was added into 200 µL of thrombin (10 nM). G-APT-Th was used as a negative control and Au-Th-Au (10 nM) solution (with or without sonication) was employed. The initial rate of scattering increase represented the relative thrombin-inhibition strength of the tested sample. Initial rates were calculated from the linear range of the early slope of the scattering profile^[3]^.

Light microscopy observation of fibrin fibers

10 µL of thrombin, G-APT-Th as a negative control and Au-Th-Au (10 nM) with or without sonication treatment were mixed with 3 µL of fibrinogen (23 µM). Subsequently, 10 µL of the mixture were transferred to a clean silicon wafer, which was covered carefully with a coverslip and allowed to dry for 3 h. After that, the different samples were analyzed by light microscopy(Leica, DMi8).

Catalytic reaction of 4-nitrophenol

Briefly, various concentrations (0-50 nM) of nanodimers solution were incubated with 4-nitrophenol (375 µM) and NaBH_4_ (100 mM) to ascertain the concentration for inducing the color change from yellow to transparent solution. Then, fibrinogen was mixed thoroughly with Au-Th-Au (50 nM) in Milli-Q water at room temperature for 5 min. The prepared mixture of fibrinogen and AuNPs was reacted with naked thrombin or G-APT-Th and Au-Th-Au (with or without US treatment) at room temperature for 20 min. A mixture containing 4-nitrophenol (600 µL) and freshly prepared NaBH_4_ was then transferred to the above solution and mixed sufficiently. After 30 min reaction, the mixture was centrifuged (12000 rpm) to remove sediment. The supernatant was transferred into a plate and absorption spectra were recorded using a plate reader (SpectraMax iD3 microplate reader, Molecular Devices).

Statistical analysis

All experiments were carried out in triplicate unless otherwise indicated. Error bars represent standard deviations. Data are presented as mean value ± SD from three independent measurements. Analysis was performed using Origin software (version: 2018, OriginLab Inc., USA). The number of samples for each analysis was introduced in each figure legend. Oneway ANOVA with Tukey’s post hoc test was used to analyse statistical differences unless specifically mentioned, ns (not significant), *P < 0.05, **P < 0.01, ***P < 0.001.

**Supplemental figures**


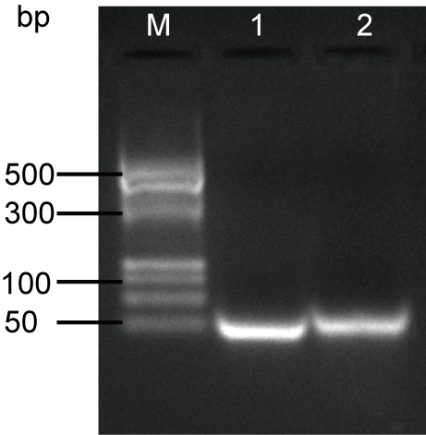


**Figure S1**. Characterization of agarose gel electropherogram of G-quadruplex aptamer. Lane M: Marker. Lane 1: G-quadruplex structure formation after annealing. Lane 2: G-quadruplex aptamer without annealing.


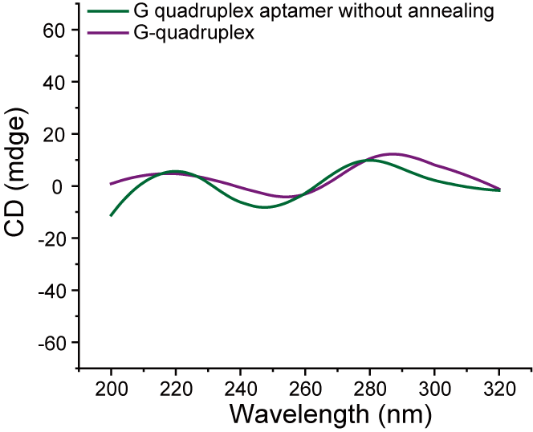


**Figure S2.** CD spectra of G-quadruplex and G-quadruplex aptamer without annealing.


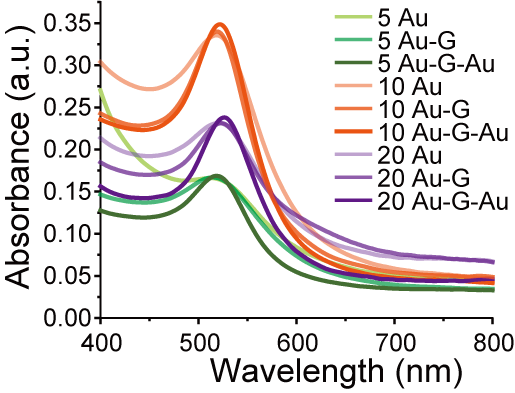


**Figure S3.** UV-vis spectra of different-sized Au, Au-G and Au-G-Au.


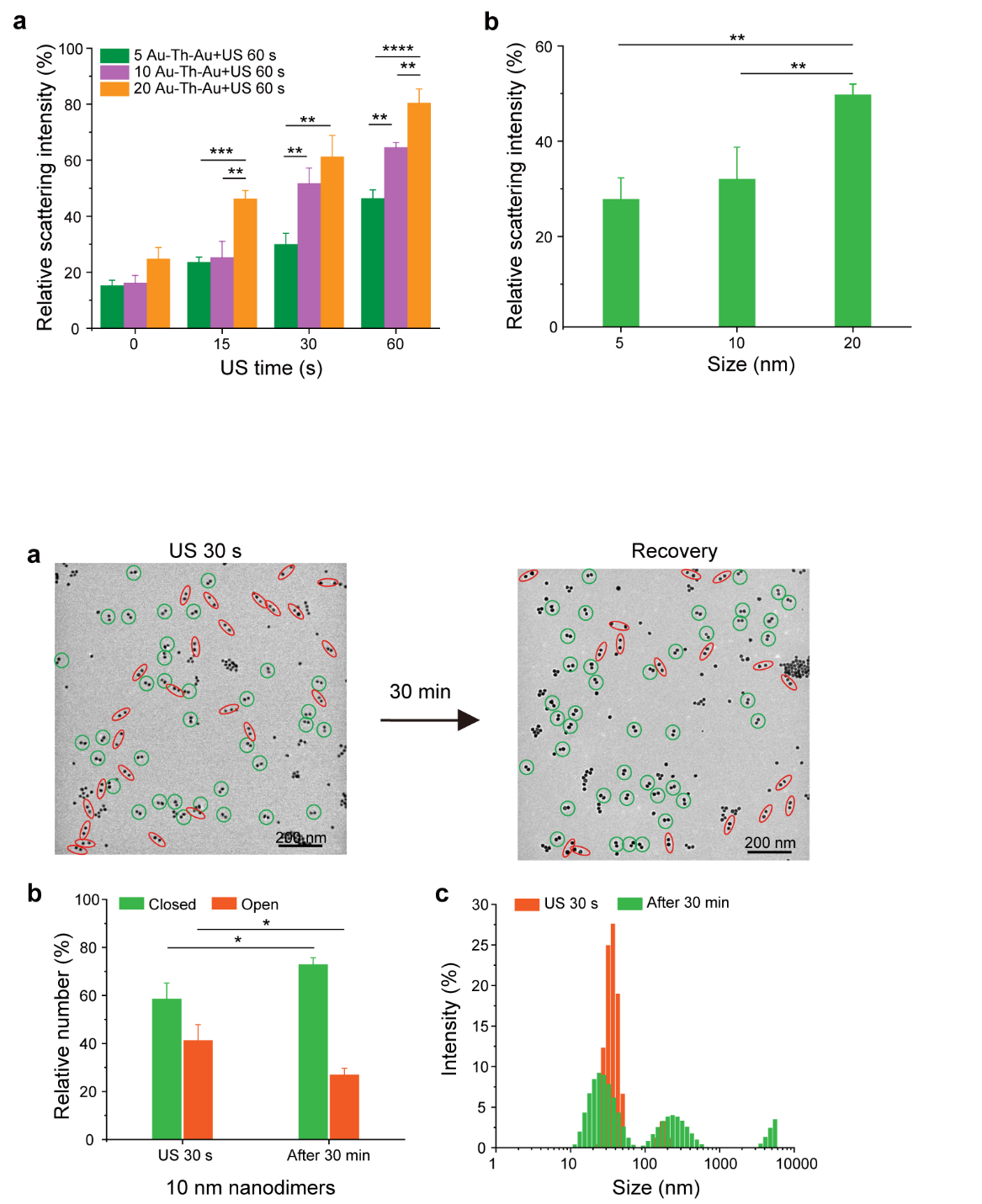


**Figure S4.** Reversibility observation of the US-triggered nanodimers. (a) Representative TEM images of Au-G-Au with US for 30 s and 30 min recovery (green circled: closed; red circled: open). Scale bars: 200 nm. (b) The relative number percentage histograms of dimer (closed), dimer (open) with US for 30 s and 30 min recovery. (c) The hydrodynamic size analysis of dimer (closed), dimer (open) with US for 30 s and 30 min recovery. The statistical analysis was repeated with three independent samples. Mean values: SD from the mean, N=3 independent experiments. Statistical differences were determined by an oneway ANOVA with Tukey’s post hoc test, *P < 0.05.


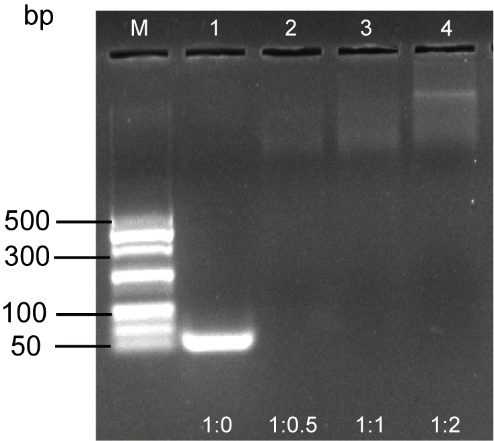


**Figure S5.** Agarose gel (4%) characterization of binding complex between G‑quadruplex and thrombin. Lane M: DNA marker; Lane 1-4: ratios of G-quadruplex to thrombin were 1:0, 1:0.5, 1:1 and 1:2, respectively.


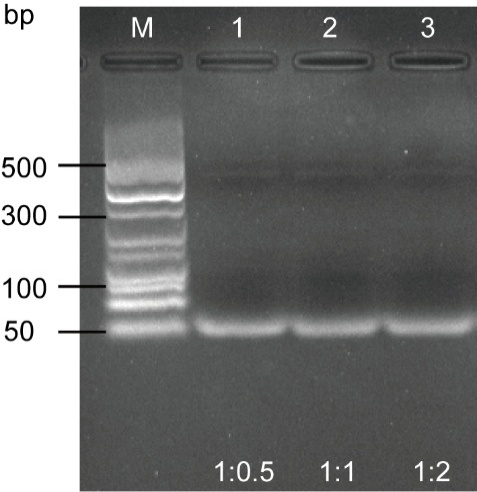


**Figure S6.** Agarose gel (4%) characterization of binding complex between G-quadruplex aptamer without annealing and thrombin. Lane M: DNA marker; Lane 1-3: ratios of G-quadruplex aptamer without annealing to thrombin were 1:0.5, 1:1 and 1:2, respectively.

**
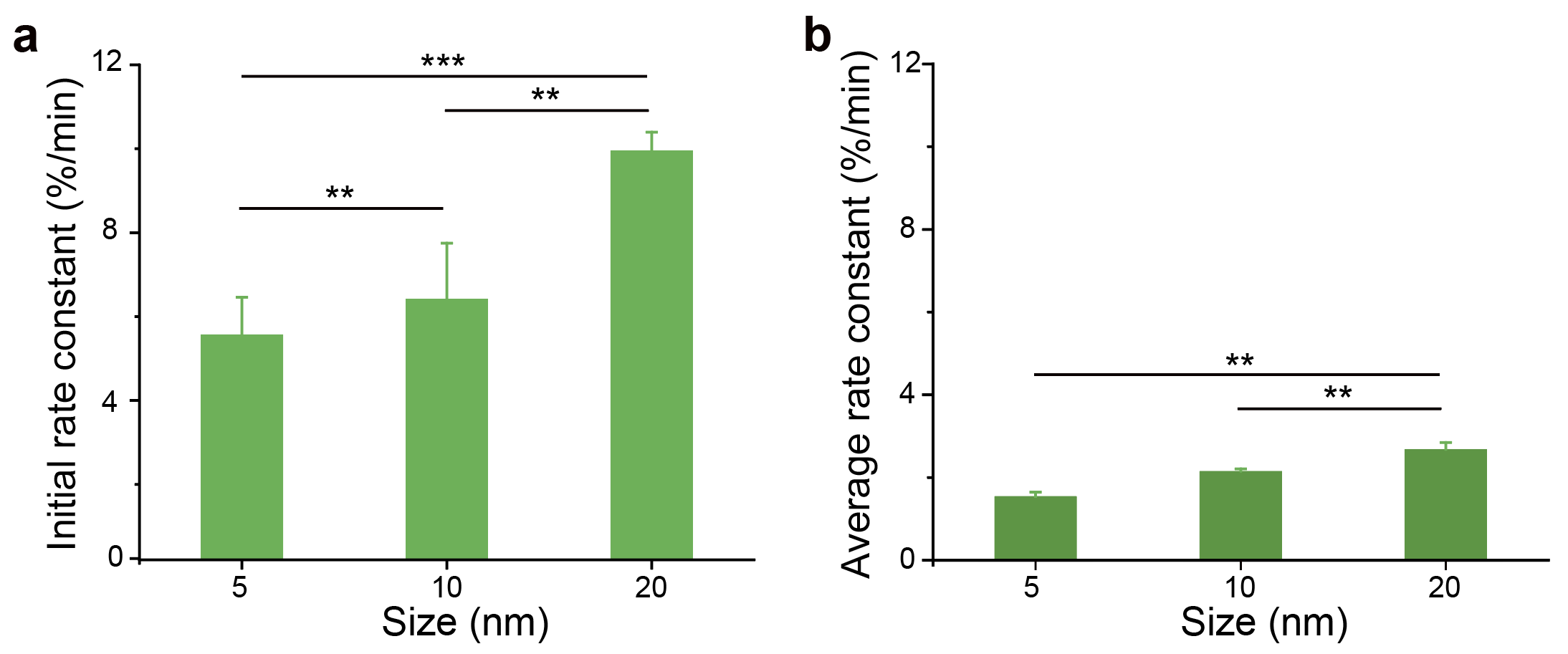
**

**Figure S7.** Rate constant of the initial rate constant (a) in the first 5 min and (b) the average rate throughout the coagulation process. Mean values ± standard deviation, N = 3 independent experiments. Statistical differences were determined by an oneway ANOVA with Tukey’s post hoc test, **P < 0.01, ***P < 0.001.

**
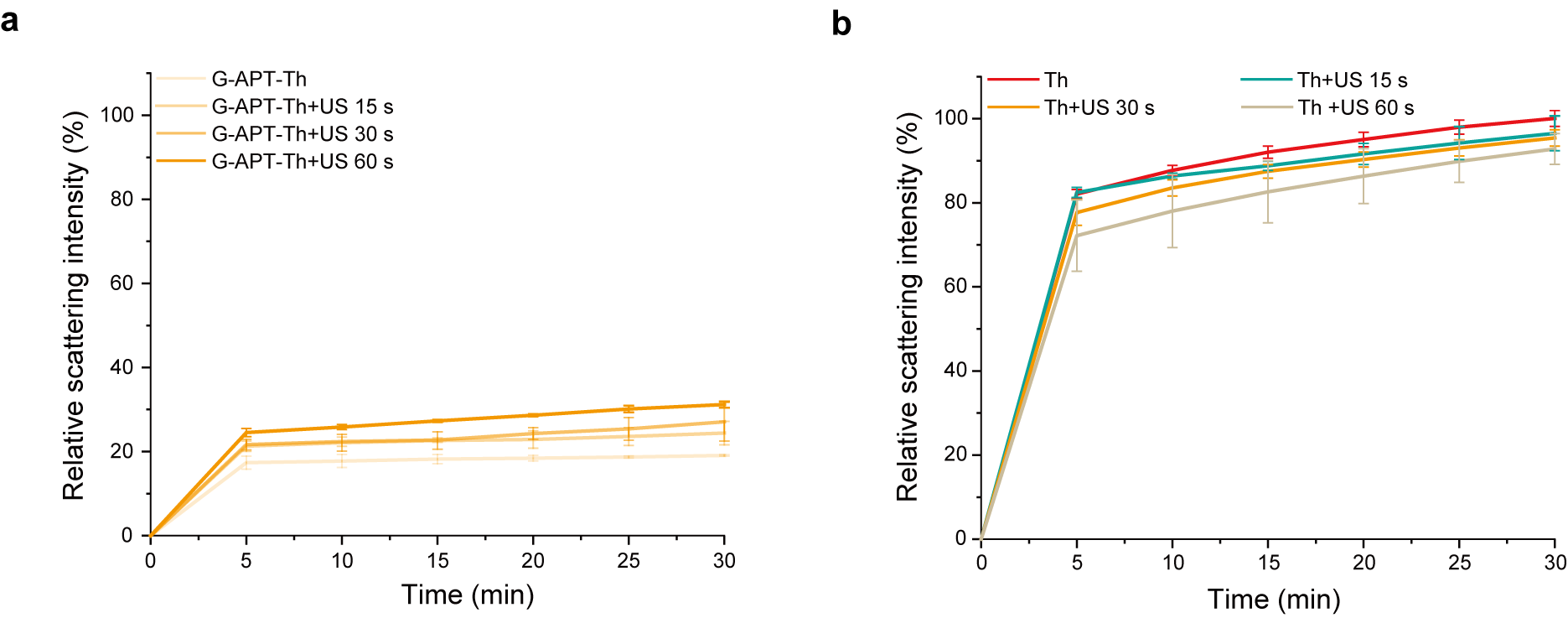
**

**Figure S8.** Real-time light scattering spectra of fibrinogen solution with added G‑APT‑Th and free thrombin with ultrasonication for 0 s, 15 s, 30 s, and 60 s, respectively. (a) G-APT-Th. (b) Thrombin. Mean values: SD from the mean, N=3 independent experiments.

**
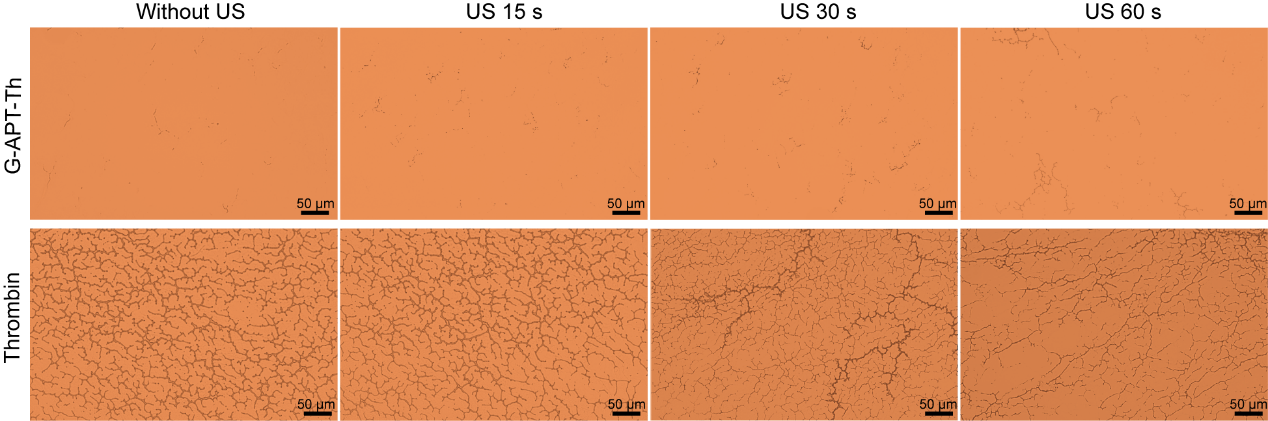
Figure S9.** Optical microscopy images of fibrinogen treated with G-APT-Th and free thrombin exposed to US for different times, respectively. Scale bars: 50 µm.


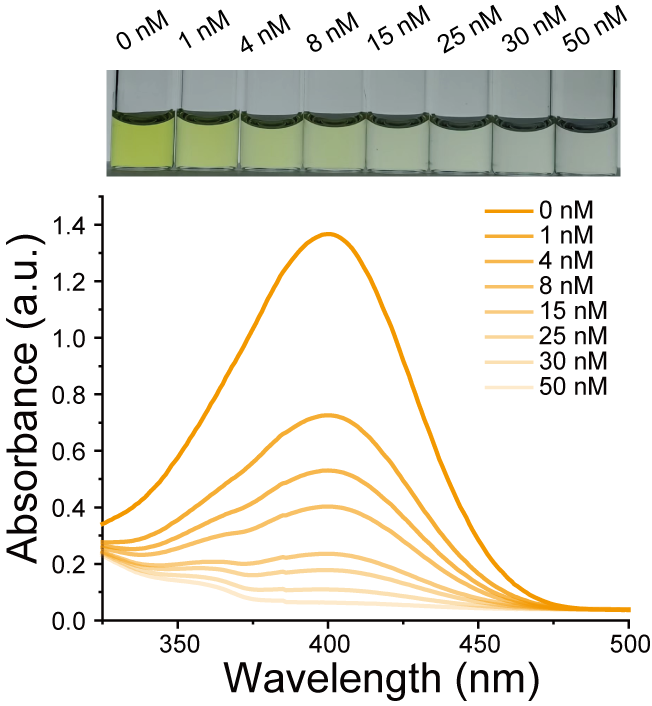


**Figure S10.** Color change and absorption spectra of 4-nitrophenol with different concentrations of 10 nm nanodimer.


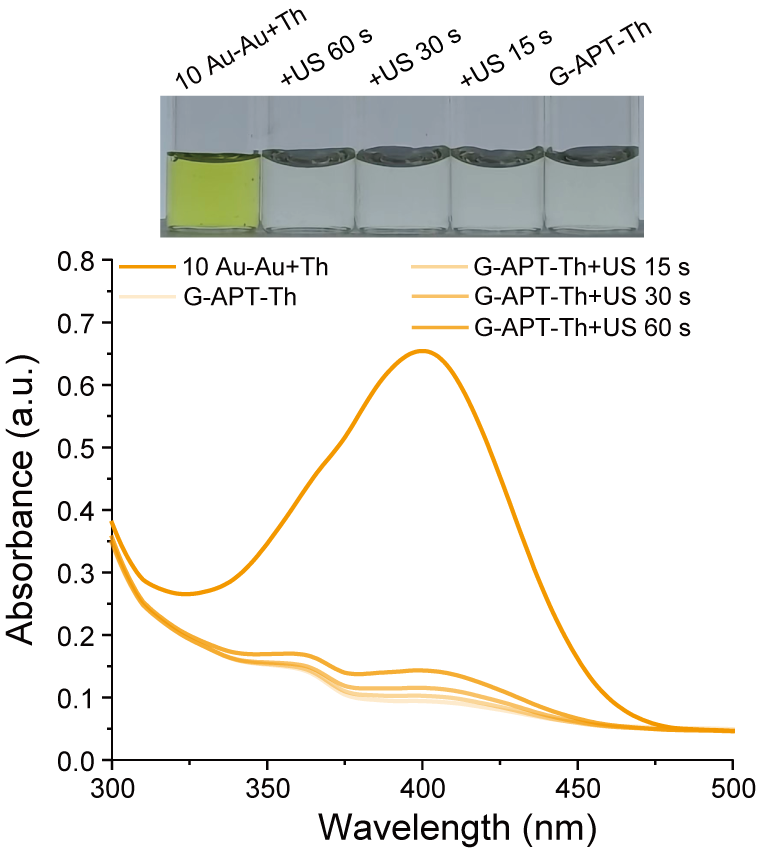


**Figure S11.** The influence of G-APT-Th on the catalytic reduction of 4-nitrophenol after different US times.

**
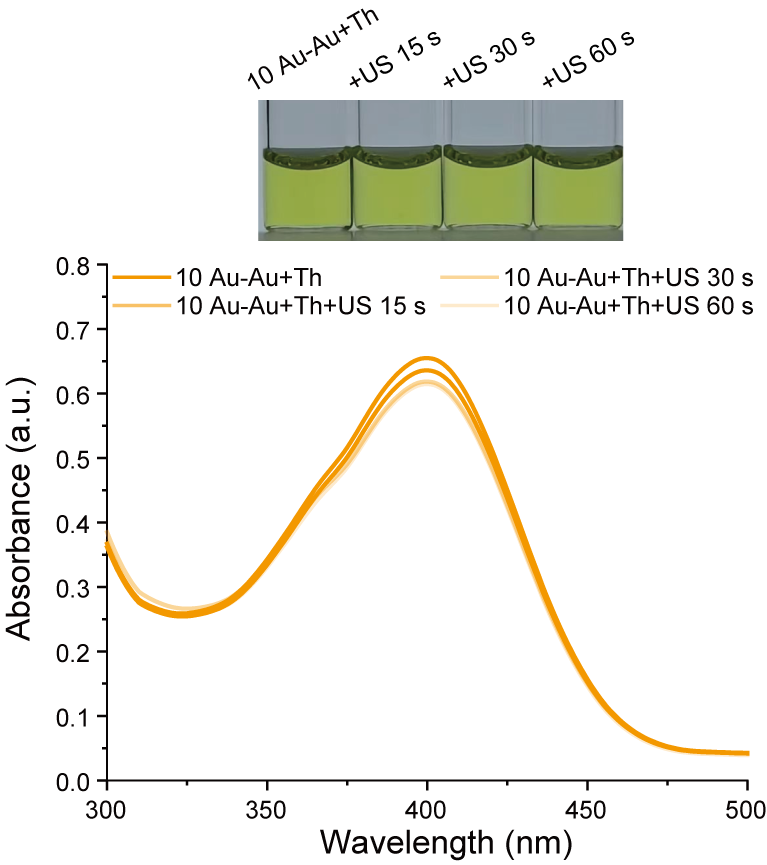
**

**Figure S12.** The influence of free thrombin on the catalytic reduction of 4-nitrophenol after different US times.

**References**

[1] H. Wang, Y. Li, M. Liu, M. Gong, Z. Deng, *Small* **2015**, *11*, 2247-2251.

[2] K. J. Neaves, J. L. Huppert, R. M. Henderson, J. M. Edwardson, *Nucleic Acids Res.* **2009**, *37*, 6269-6275.

[3] Y. Kim, Z. Cao, W. Tan, *Proc. Natl. Acad. Sci. U. S. A.* **2008**, *105*, 5664-5669.
